# Coarse spatial resolution remote sensing data with AVHRR and MODIS miss the greening area compared with the Landsat data in Chinese drylands

**DOI:** 10.3389/fpls.2023.1129665

**Published:** 2023-05-17

**Authors:** Jianshuang Zhang, Yangjian Zhang, Nan Cong, Li Tian, Guang Zhao, Zhoutao Zheng, Jie Gao, Yixuan Zhu, Yu Zhang

**Affiliations:** ^1^ Key Laboratory of Ecosystem Network Observation and Modeling, Institute of Geographic Sciences and Natural Resources Research, Chinese Academy of Sciences, Beijing, China; ^2^ College of Resources and Environment, University of Chinese Academy of Sciences, Beijing, China; ^3^ CAS Center for Excellence in Tibetan Plateau Earth Sciences, Chinese Academy of Sciences, Beijing, China; ^4^ University of Chinese Academy of Sciences, Beijing, China

**Keywords:** vegetation greening, NDVI, ecological engineering projects, Google Earth Engine, Chinese drylands

## Abstract

The warming-wetting climates in Chinese drylands, together with a series of ecological engineering projects, had caused apparent changes to vegetation therein. Regarding the vegetation greening trend, different remote sensing data had yielded distinct findings. It was critical to evaluate vegetation dynamics in Chinese drylands using a series of remote sensing data. By comparing the three most commonly used remote sensing datasets [i.e., MODIS, Advanced Very High Resolution Radiometer (AVHRR), and Landsat], this study comprehensively investigated vegetation dynamics for Chinse drylands. All three remote sensing datasets exhibited evident vegetation greening trends from 2000 to 2020 in Chinese drylands, especially in the Loess Plateau and Northeast China. However, Landsat identified the largest greening areas (89.8%), while AVHRR identified the smallest greening area (58%). The vegetation greening areas identified by Landsat comprise more small patches than those identified by MODIS and AVHRR. The MODIS data exhibited a higher consistency with Landsat than with AVHRR in terms of detecting vegetation greening areas. The three datasets exhibited high consistency in identifying vegetation greening in Northeast China, Loess Plateau, and Xinjiang. The percentage of inconsistent areas among the three datasets was 39.56%. The vegetation greening areas identified by Landsat comprised more small patches. Sensors and the atmospheric effect are the two main reasons responsible for the different outputs from each NDVI product. Ecological engineering projects had a great promotion effect on vegetation greening, which can be detected by the three NDVI datasets in Chinese drylands, thereby combating desertification and reducing dust storms.

## 1 Introduction

China is home to 6.6 million km^2^ of drylands, which support approximately 580 million people (Li et al., 2021). Distributed from east to west in northern China, the drylands of China act as an ecological barrier to China, playing a critical role in protecting biodiversity and providing ecosystem services (Poulter et al., 2014; Huang et al., 2017; Mao et al., 2018). Dryland ecosystems are also vital to the global carbon cycle, dominating the interannual variability of global terrestrial ecosystem carbon (Poulter et al., 2014; Ahlstrom et al., 2015; Biederman et al., 2017). Climates in Chinese drylands have changed in a unique pattern in recent years. Together with warming, it has gradually become wetter since the 1960s (Song and Zhang, 2003; Shi et al., 2007; Chen and Dai, 2009; Han et al., 2016; Li et al., 2016; Peng and Zhou, 2017), especially transiting from warm-dry to warm-wet since the mid-1980s (Shi et al., 2003). The “warming-wetting” climate has favored greening in Northwest China (Zhao et al., 2011; Fang et al., 2013; Liu et al., 2018; Wu et al., 2019; Wang et al., 2020), which is further boosted by a series of national ecological engineering projects (Chen et al., 2019), including the Three Norths Shelter Forest System Project (TSFP), the Natural Forest Conservation Program (NFCP), the Grain to Green Program (GTGP), the Sand Control Programs surrounding Beijing and Tianjin (BSCP), and the conservation and restoration project in the Three-River Source Region (TSRP) (Liu et al., 2008; Shao et al., 2017). China has the world’s largest afforested area with ∼62 million hectares in 2008, which is expected to increase by approximately 40 million hectares from 2005 to 2020 (Peng et al., 2014). These ecological engineering projects promoted vegetation greening, enhanced ecosystem carbon sequestration, and reduced land desertification (Tong et al., 2018).

Vegetation greening, the interannual increasing trends of vegetation greenness, has occurred widely on a regional to global scale (Chen et al., 2021; de Jong et al., 2012; Zhu et al., 2016; Choler et al., 2021; Zhao et al., 2022). The greening of the Earth’s land has been accelerating since the early 1980s, and China is the primary contributor to global greening and accounts for over 25% of the global net increase in leaf area (Mao et al., 2016; Chen et al., 2019; Cortes et al., 2021). Various remote sensing data all reported that China has experienced an unambiguous greening since 1982 (Piao et al., 2003). Climate change, rising atmospheric CO_2_ concentration, nitrogen deposition, and some positive human interventions all contribute to Chinese land greening (Donohue et al., 2013; Keenan et al., 2016; Naeem et al., 2021; Berdugo et al., 2022; Huang et al., 2016; Gao et al., 2022; Peng et al., 2010; Piao et al., 2013; Piao et al., 2015).

The normalized difference vegetation index (NDVI) is the most commonly used remote sensing index in monitoring vegetation dynamics due to its numerous advantages, such as wide space-time coverage and high sensitivity to vegetation coverage changes (Guo et al., 2018; Zhang et al., 2021). Among the ensemble, the Advanced Very High Resolution Radiometer (AVHRR) sensors represent the longest continuous data series (Tucker et al., 2005), and the MODIS vegetation index (VI) represents higher spatial resolution and an intensively validated product (Zhang et al., 2017). Aside from these two datasets, Landsat with higher spatial resolution can generate time series vegetation dynamics, too. However, each dataset has yielded inconsistent findings (Zhang et al., 2013). In the meantime, their different spatial resolutions underscore their distinct capability in detecting vegetation dynamics under disparate background contexts. The vegetation in arid and semiarid regions is naturally sporadically distributed and grows with low coverage. These growth statuses pose a great challenge for remote sensing data in monitoring vegetation changes. Coarse spatial resolution data might miss some patchy vegetation greening, while high spatial resolution data with low temporal resolution are constrained by its intermittent data availability. Subpixel spatial heterogeneity of vegetative greening and browning is hardly expressed in coarse spatial resolution data (Myers-Smith et al., 2020). Datasets with increased spatial–temporal resolutions can reduce false vegetation changes caused by their own error of low resolution. One major explanation for the many inconsistencies in our current understanding of plant dynamics in Chinese drylands is the use of various remote sensing data. The sensors themselves and the atmospheric effect (Fan and Liu, 2016), the red band error of atmospheric effect (Guo et al., 2017), the data extraction method (Donohue et al., 2008; Zhang et al., 2013) and different scales, other factors such as the sun’s elevation angle, the spectral characteristics of the sensor band center and bandwidth, the inconsistency of atmospheric quality, and the different surface vegetation coverage may lead to inconsistent spatial changes of vegetation in different remote sensing datasets (Zhang et al., 2020; Liu et al., 2022).

To achieve uniformity in research findings from each study and advance our knowledge on vegetation dynamics in Chinese drylands, we better compare the performances of each data. In this study, the three commonly used datasets with various spatial resolutions are processed on the Google Earth Engine platform (Gorelick et al., 2017), and their performances are compared in detecting vegetation greenness in Chinese drylands. Our threefold objectives are to 1) evaluate where and how Chinese drylands have become greening, 2) compare the greening or browning trend areas detected by the three datasets, and 3) identify the regions where the three datasets have the greatest consistency or discrepancies. The research findings can deepen our knowledge on vegetation greenness dynamics in Chinese drylands under the warming-wetting climate trends.

## 2 Materials and methods

### 2.1 Study area

**Figure 1:**
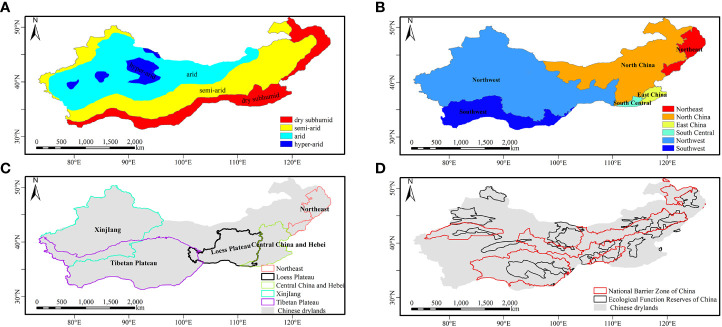
The study area in Chinese drylands. **(A)** Distribution of dryland type; **(B)** distribution of geographical regions; **(C)** distribution of typical regions; **(D)** distribution of ecological engineering projects.

The study area encompasses Chinese drylands, which mainly include the Inner Mongolia Plateau, the Loess Plateau, and the Tibetan Plateau (**Figure 1**). The boundaries of the study area and aridity zones are based on previously established standards (**Figure 1A**) (Huang et al., 2017; Zhu et al., 2022). The ranges of the Ecological Function Reserves of China (https://www.resdc.cn/data.aspx?DATAID=137) and the National Barrier Zone of China (http://www.geodoi.ac.cn/WebCn/doi.aspx?Id=1453) were also used in this study (**Figure 1D**).

### 2.2 Data

The Google Earth Engine (GEE) provides online access to archived MODIS/AVHRR/Landsat NDVI data (**Table 1**). This study selected MOD13Q1 V6 (Didan, 2015) NDVI products with a temporal resolution of 16 days and a spatial resolution of 250 m. MOD13Q1 had applied atmospherically corrected bidirectional surface reflectance to NDVI products on GEE, thus having low clouds and view angle effects. The NOAA Climate Data Record (CDR) of AVHRR NDVI is a daily product derived from the NOAA AVHRR Surface Reflectance at a spatial resolution of 0.05° (Vermote and NOAA CDR Program, 2019).

**Table 1:** The NDVI datasets and climate data archive on the GEE.

Data	Dataset provider	Resolution
NOAA CDR AVHRR NDVI: Version 5	NOAA	0.05°
MOD13Q1.006	USGS EROS Center	250 m
Landsat5/7/8 Collection 2 Surface Reflectance	USGS	30 m

Landsat Collection 2 marks the second major reprocessing effort on the Landsat archive by the USGS that resulted in several data product improvements over Collection1 by harnessing recent advancements in data processing and algorithm development. This study used the Landsat Collection 2 Tier-1 surface reflectance (SR) data on the Google Earth Engine website, including Landsat 5 TM, Landsat 7 ETM, and Landsat 8 OLI for Red and NIR bands. The Landsat collection was atmospherically corrected and ortho-rectified, and the annual median NDVI from June to September was utilized in this study.

### 2.3 Method

The annual maximum NDVI composited for all MODIS and AVHRR NDVI available on the GEE from 2000 to 2020 was extracted for Chinese drylands. Sen’s slope estimator was applied to detect vegetation change trends. The SEN (Sen, 1968) trend analysis and the Mann–Kendall (MK) test (Mann, 1945; Kendall, 1957) were widely used in meteorological and hydrological research studies (Gocic and Trajkovic, 2013). The prior one was more suitable for studying vegetation change trends than linear regression methods (Fensholt et al., 2012). The latter one was used to determine the NDVI’s trend significance (Li et al., 2019). The trend of NDVI was divided into four classes: significant greening (*ρ* > 0, | *Z* | > 1.96), significant browning (*ρ* < 0, | *Z* | > 1.96), non-significant greening (*ρ* > 0, | *Z* | ≤ 1.96), and non-significant browning (*ρ* < 0, | *Z* | ≤ 1.96). The *ρ* was the Sen slope’s slope. If the MK test statistics (| *Z* |) was greater than 1.96, it was considered to have passed the significance test of 95%.

## 3 Result

### 3.1 Spatial analysis and regional statistics

**Figure 2:**
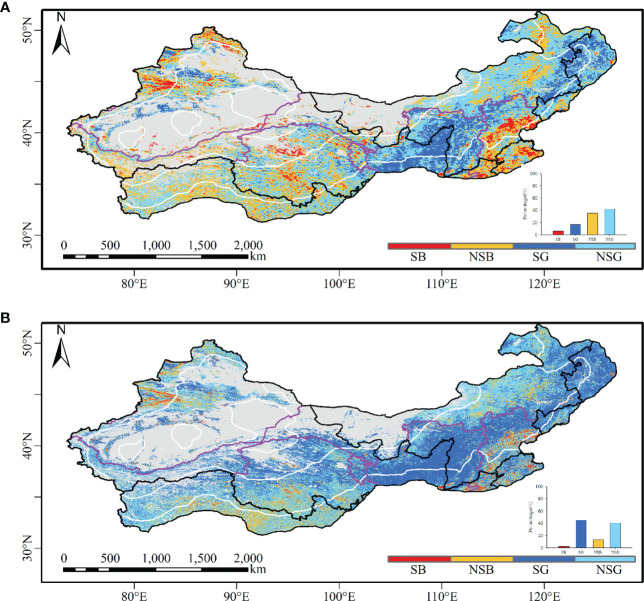
The spatial distribution of vegetation greening and browning from the AVHRR and MODIS NDVI datasets. **(A)** Vegetation coverage trends based on the AVHRR; **(B)** vegetation coverage trends based on MODIS. SB, significant browning; NSB, non-significant browning; SG, significant greening; NSG, non-significant greening.

Based on AVHRR (**Figure 2A**), the vegetation non-significant browning areas were mainly distributed in the Tibetan Plateau and the northern part of North China. The significant vegetation browning areas were primarily located in South Central and East China, the southern part of North China, and some scattered regions in western China. The vegetation non-significant greening areas were mainly distributed in North and Northeast China and a few scattered patches in Northwest China. The areas with significant vegetation greening were primarily distributed in the Loess Plateau and Northeast China and a few scattered patches in Northwest China.

The MODIS data identified obviously larger greening areas than the AVHRR data (**Figure 2**). Based on the MODIS data (**Figure 2B**), the vegetation non-significant browning areas were mainly distributed in the southern Tibetan Plateau and the northern and southern parts of North China. The areas with significant vegetation browning were primarily distributed in South Central and East China, the southern part of North China, and the northwestern part of Chinese drylands. The vegetation non-significant greening areas based on the MODIS dataset were mainly distributed in the southern Tibetan Plateau, the northern part of North China, and the northern part of northwestern China. The significant vegetation greening areas were primarily located in the Loess Plateau, Northeast China, and the northern Tibetan Plateau.

The vegetation greening areas identified by MODIS comprised more small patches than those identified by AVHRR. The greening areas identified by AVHRR were smaller than those identified by MODIS, while the browning areas identified by AVHRR were larger than those identified by MODIS. The sharp distinction between AVHRR and MODIS in identifying vegetation non-significant greening occurred mostly in western China (**Figure S1**). Meanwhile, apparent differences existed in the northern part of Northwest China and the northern Tibetan Plateau, where vegetation turned significantly brown based on AVHRR but turned significantly green based on MODIS. The non-significant greening areas exhibited the highest consistency between AVHRR and MODIS, followed by significant vegetation greening, vegetation non-significant browning, and significant vegetation browning. The consistent non-significant browning areas between AVHRR and MODIS were 290,645.1 km^2^, which were mostly distributed in the southern Tibetan Plateau, South Central China and East China, and some parts of North China. The consistent areas with significant browning areas were 21,573.17 km^2^, mainly concentrated in South Central China and East China, the southern parts of North China, and a few scattered patches in northwestern China. The consistent areas with vegetation non-significant greening were 1,523,174.27 km^2^, mainly distributed in the northern part of North China and the Tibetan Plateau. The consistent areas with significant greening areas were 480,836.24 km^2^, mainly distributed in the Loess Plateau and Northeast China.

For the entire study area, the vegetation greening area was larger than that of vegetation browning (**Figure S2**). For both the AVHRR and MODIS data, the significant vegetation greening areas were the largest in the semiarid regions, followed by dry subhumid, arid, and hyperarid regions. AVHRR revealed non-significant greening with 52.84% and 59.29% in the hyperarid and arid regions of the study area, respectively, while MODIS identified 72.65% and 49.51% of the two regions as significant greening, respectively. In the semiarid and dry subhumid regions, the dominant change was non-significant greening based on the AVHRR data, while it changed to significant greening based on MODIS. The area proportion was the lowest for significant browning based on the AVHRR data in semiarid regions, whereas the same index for the MODIS data was the lowest in hyperarid regions. Vegetation had turned significantly green as a whole in the Chinese drylands. The area with identical changes between AVHRR and MODIS was the largest for non-significant greening, followed by significant greening, non-significant browning, and significant browning areas in Chinese drylands.

With regard to geographical regions (**Figure S3**), vegetation showed greening trends in 50.03%, 51.74%, and 40.25% of the areas in Northeast, Northwest, and North China based on AVHRR, respectively, while MODIS changed to significant greening with 70.59%, 48.00%, and 48.00%, respectively. The significant vegetation greening areas with MODIS were larger than those with AVHRR, and the vegetation non-significant greening areas were also larger than the vegetation non-significant browning areas for both AVHRR and MODIS datasets in Chinese drylands. Among the six geographical regions, the proportion of non-significant browning areas was the lowest in Northeast China for the AVHRR as well as MODIS data. In Southwest China, vegetation non-significant greening area accounted for 48.91% and 54.42% based on AVHRR and MODIS, respectively. In East China and South Central China, vegetation non-significant browning identified by the AVHRR data accounted for 50.34% and 53.42% of the region, respectively, while the region turning significantly green covered small areas. With the MODIS data, vegetation non-significant greening occurred in 39.22% and 34.71% of the same region, respectively, which was similar to the significant greening areas. In Northwest, Northeast, and North China, the identical trends from the AVHRR and MODIS data had the largest non-significant greening trends, followed by significant greening, non-significant browning, and significant browning. The pattern in Southwest China was similar to that in Central South China, with the largest area of vegetation non-significant greening, followed by non-significant browning, significant greening, and significant browning, but Central South China displayed an overall non-significant browning trend. Different from other regions, East China displayed that the area with identical patterns between the two datasets was the largest for vegetation non-significant greening, followed by non-significant browning, significant browning, and significant greening. Overall, the consistency between the two datasets was higher in Northwest China than in North China, while these two regions had a higher consistency than other geographical regions in Chinese drylands.

### 3.2 Comparison of MODIS and AVHRR products with Landsat data

**Figure 3:**
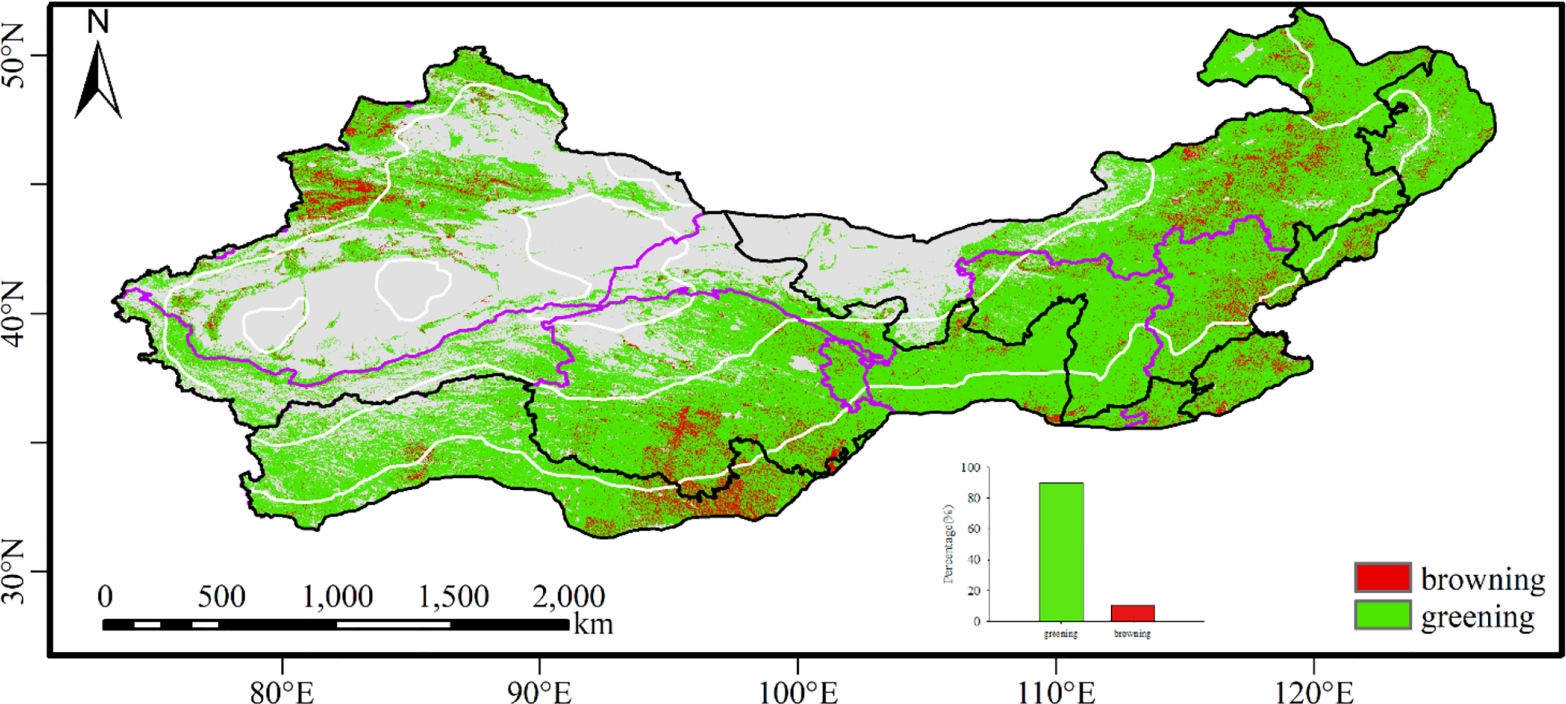
The spatial distribution of vegetation greening and browning with Landsat data from 2000 to 2020.

**Figure 4:**
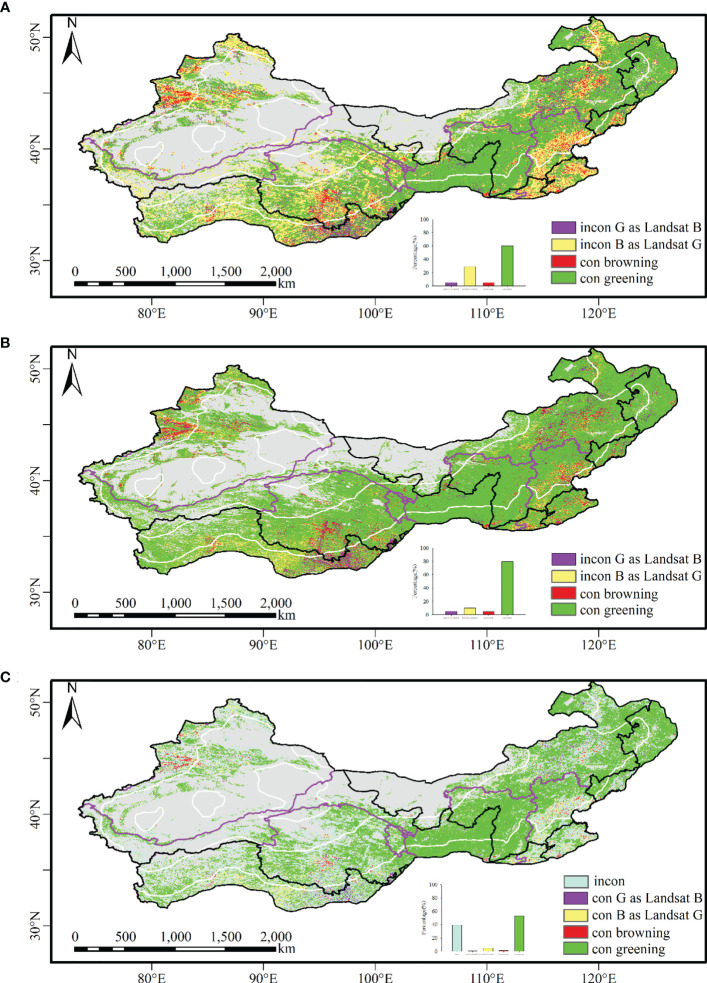
The spatial distribution of consistent and inconsistent regions of Landsat with AVHRR and MODIS NDVI datasets from 2000 to 2020. **(A)** Comparison between Landsat and AVHRR; **(B)** comparison between Landsat and MODIS; **(C)** comparison among Landsat, MODIS, and AVHRR.

Landsat identified larger greening areas (89.8%) than MODIS as well as AVHRR (58%) (**Figures 3**, **S4**). Regarding identifying vegetation change, Landsat showed higher consistency with MODIS data than with AVHRR (**Figure 4**). The consistency region with the Landsat and AVHRR data was broadly similar to the Landsat and MODIS data, but the areas that turned green were smaller with the Landsat and AVHRR data (**Figures 4A, B**). Landsat and MODIS had high consistency in identifying vegetation greening in Northeast China, the Loess Plateau, and Xinjiang and in identifying vegetation browning in the southeastern part of the Tibetan Plateau, parts of Xinjiang, and some parts of North China (**Figure 4B**). The regions with high inconsistency between the AVHRR and Landsat data were located in the Tibetan Plateau and parts of Central China and Hebei Province, which were identified as browning by AVHRR but greening by Landsat data. The areas with large differences between MODIS and Landsat were mainly located in the southeast of the Tibetan Plateau, parts of Central China and Hebei Province, and near the regions of consistent vegetation browning in the northern part of North China.

The three datasets exhibited high consistency in identifying vegetation greening in Northeast China, the Loess Plateau, and Xinjiang, while their consistency was the lowest on the Tibetan Plateau, especially the southern part of the Tibetan Plateau (**Figures S4**, **4**). The percentage of consistent vegetation browning areas with the three datasets was 1.36%, mainly distributed in the southeastern part of the Tibetan Plateau and parts of Xinjiang. The percentage of areas that were identified as browning by AVHRR and MODIS but greening by Landsat was 4.89%, which was primarily located in the central part of Southwest China and parts of Xinjiang. The consistent vegetation greening areas by AVHRR and MODIS but browning by Landsat were mainly located in the southeastern part of the Tibetan Plateau, with a percentage of 0.87%.

The percentage of inconsistent areas among the three datasets was 39.56%, which was mainly distributed in the southern part of the Tibetan Plateau, the northern part of North China, and Central China and Hebei Province. Meanwhile, the area of vegetation greening identified by MODIS and Landsat, but browning by AVHRR, accounts for 50.50% of the inconsistent areas, while the vegetation greening identified by AVHRR and Landsat, but browning by MODIS, accounts for 9.85% of the inconsistent areas.

To further compare the performance between AVHRR and MODIS data, we generated Sen’s slope for the three datasets and then resampled Landsat data to spatial resolutions of 5,000 and 250 m to match AVHRR and MODIS data, respectively (**Figure S5**). The *R* ^2^ for the correlation analysis between AVHRR and MODIS with Landsat was 0.1257 and 0.3983, respectively.

## 4 Discussion

Generally speaking, the areas of vegetation browning and significant browning with AVHRR data were larger than those with MODIS data, while the significant vegetation greening areas with AVHRR data were smaller than those with MODIS (**Figures S2**, **S3**). A similar pattern was also identified in several typical regions with large-scale ecological engineering projects, such as the Loess Plateau and Northeast China (**Figure S4**). From this, we can conclude that MODIS has identified a larger area of significant vegetation greening. Landsat has generally the most vegetation greening areas, followed by MODIS and AVHRR. Coarse resolution data might miss some land use and land cover changes.

### 4.1 Reasons for the differences among the AVHRR, MODIS, and Landsat NDVI datasets

Sensors and the atmospheric effect are the two main reasons responsible for the different outputs from each NDVI product (Nemani et al., 2003; Fan and Liu, 2016). The red band error due to atmospheric effects is one of the main sources (Guo et al., 2017). AVHRR NDVI has been reported to be biased in generating time series due to sensor degradation (Tarnavsky et al., 2008; Tian et al., 2015). The AHHRR sensors are updated more frequently than those of MODIS, and the bandwidths of each sensor are adjusted accordingly (Zhang et al., 2015). In monitoring vegetation dynamics, MODIS NDVI loaded with an advanced navigation system and improved radiometric sensitivity is considered to be superior to AVHRR-based NDVI (Huete et al., 2002; Fensholt and Proud, 2012). The spectral ranges of the MODIS red band with 620–670 nm and near-infrared bands with 841–876 nm are narrower than those of AVHRR (Gallo et al., 2005), and the prior one can eliminate water absorption effects to some extent (Liu et al., 2022). MODIS C5 sensor degradation can also affect its time series comparability and cause a fake vegetation browning trend (Wang et al., 2012). Compared with AVHRR and MODIS, Landsat Collection 2 data provide well-calibrated, precisely geolocated, and improved spectral coverage images and apply geometric and radiometric processing to acquire a seamless effect among multisensors, which improves the interoperability of images through time (Wulder et al., 2022); therefore, the observed satellite changes can be attributed to surface changes rather than to instrument changes (Markham and Helder, 2012; Cohen et al., 2016). Meanwhile, cloud effects can further introduce some uncertainties, which can affect the retrieval accuracy of coarse resolution images (Alcaraz-Segura et al., 2010).

**Figure 5:**
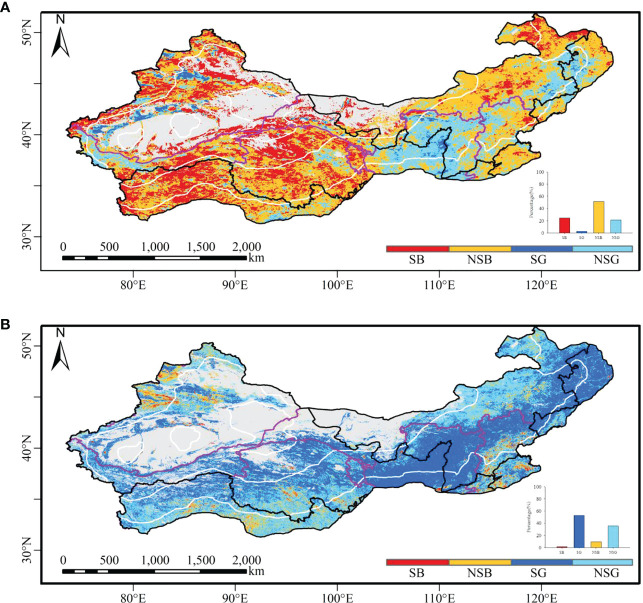
The spatial distribution of vegetation greening and browning with the NDVImedian from June to September for the two datasets. **(A)** AVHRR greening; **(B)** MODIS greening.

Although the new versions of MODIS and AVHRR have improved their product quality (Zhang et al., 2017; Liu et al., 2022), the data synthesis process can cause information loss (Alcaraz-Segura et al., 2010). This study uses the maximum value composition method for the two coarse spatial resolution data. We further compare its performance with the median value composite method. According to the median value composite method, the vegetation change pattern difference between the two datasets is even greater, especially in western China (**Figure 5**). The spatial distribution of AVHRR using NDVImedian from June to September is quite different from that of annual NDVImax (**Figures 2A**, **5A**). The significant greening areas with NDVImedian from June to September with AVHRR data were far less than those of the annual NDVImax, while the significant browning areas were larger. The spatial distribution of NDVImedian from June to September and annual NDVImax for MODIS data was roughly the same (**Figures 2B**, **5B**). The significant greening areas of the NDVImedian were slightly larger than those of the annual NDVImax, while the significant browning areas were smaller with the MODIS data.

GIMMS, MODIS, and Landsat datasets are generally more or less the same in detecting global vegetation change trends (Fensholt et al., 2012; Scheftic et al., 2014), but with different greening rates (Tian et al., 2021). The reason for the high consistency of various data in detecting vegetation greening in the Loess Plateau and Northeast China is related to the fact that these two regions have become significantly greening under a series of ecological engineering projects (Xiao, 2014; Chen et al., 2015; Guo et al., 2017; Cao et al., 2018). The region where the three datasets reveal the analogous browning pattern is mostly overlapping with fast urbanization, such as Central China and Hebei Province. In the central part of Southwest China and parts of Xinjiang, only Landsat data can identify vegetation greening. In these regions, vegetation greening has occurred in a patchy pattern, which is beyond the detection capacity of coarse spatial resolution remote sensing data. On the other hand, vegetation has become greening with AVHRR and MODIS but browning with Landsat in the eastern part of Southwest China. The reason can be related to the small number of available Landsat images due to cloudiness.

However, these three datasets turn out distinct results in some specific regions, such as the northern Tibetan Plateau, which has also been reported in previous related studies (Jiang et al., 2017; Burrell et al., 2018; Liu et al., 2022). Land use change (LUC) is one of the most influential factors in vegetation greenness (Hua et al., 2017). LUC caused by different factors can manifest in distinct patterns. In Northern China, the vegetation significant greening is due to increased fractional cover in crops, grasslands, and forests (Hua et al., 2017). In the Tibetan Plateau, vegetation greening is mostly caused by climate change (Zhu et al., 2016), while LCC mostly makes the greatest contribution to regional greening in southeast China (Zhu et al., 2016).

### 4.2 The impact of ecological engineering projects on vegetation greening

**Figure 6:**
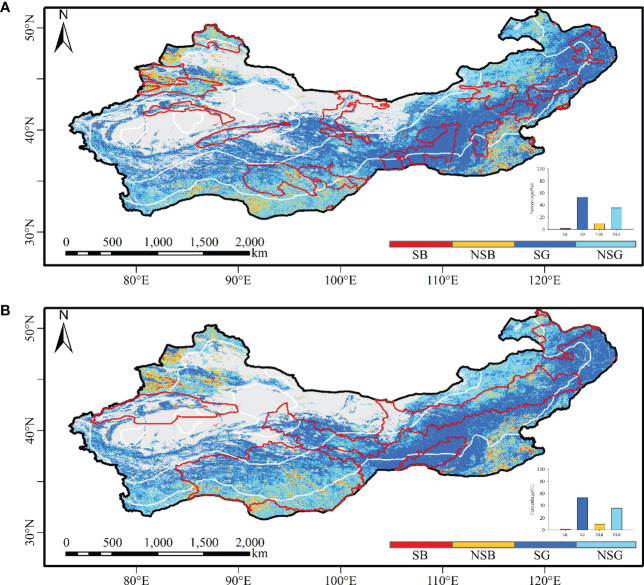
The spatial distribution of vegetation greening and browning from MODIS NDVI data in the Ecological Function Reserves and the National Barrier Zone of China. **(A)** Ecological Function Reserves of China; **(B)** the National Barrier Zone of China.

Vegetation change trends exhibit large differences among each ecological function reserve, with an overall greening pattern in the Ecological Function Reserves and the National Barrier Zone (**Figure 6**). Large-scale ecological engineering projects bring about fundamental changes to vegetation in Chinese drylands, especially in the Loess Plateau and Northeast China (Zhang et al., 2016; Yu et al., 2021). For example, the implementation of the “Grain for Green Program” (GFGP) (Song et al., 2022) ecological engineering on the Loess Plateau has significantly led to an increase in vegetation coverage (Xiao, 2014; Chen et al., 2015; Cao et al., 2018), especially in the regions where precipitation is higher than 400 mm (Li et al., 2015; Cao et al., 2018). After the implementation of the ecological project, vegetated land has increased significantly by 65.78% on the Loess Plateau (Xiu et al., 2021). Ecological engineering projects of the GFGP had a great promotion effect on vegetation greening, which can be detected by the three NDVI datasets. Meanwhile, large-scale afforestation should be implemented especially considering local conditions to strengthen ecological resilience in Chinese drylands, thereby combating desertification and reducing dust storms.

For the AVHRR, MODIS, and Landsat datasets, the vegetation turned significantly green in the regions where the transformation from grassland and cultivated land to forest land and from cultivated land to grassland was implemented, such as the Loess Plateau and Northeast China. The percentage of vegetation browning with the AVHRR, MODIS, and Landsat NDVI datasets for the National Barrier Zone of China was 33.41%, 15.01%, and 10.77%, respectively. The percentage of vegetation browning with the AVHRR, MODIS, and Landsat NDVI datasets for the Ecological Function Reserves of China was 31.52%, 16.12%, and 11.14%, respectively. It can be seen that Landsat data detected the highest vegetation greening in the ecological engineering project zone, followed by MODIS and AVHRR. For example, in the central part of Southwest China, high spatial resolution remote sensing data could identify vegetation greening, while coarse spatial resolution remote sensing datasets with AVHRR and MODIS detected vegetation browning, and the areas of browning in AVHRR data was much larger than in MODIS (**Figure 4**). The vegetation greening in these areas was more dispersed, and more greening areas were identified as the resolution increased.

By typical regions (**Figure S6**), for the Loess Plateau, AVHRR and MODIS identify significant greening in 49.80% and 78.21% of the region, respectively. In Northeast China, the AVHRR data identify non-significant greening in 49.41% of the region, while the MODIS data detect significant vegetation greening in 70.59% of the region. In some regions located in Central China and Hebei Province, the AVHRR data are mainly focused on vegetation non-significant browning (38.79%). Unlike the AVHRR data, the MODIS data are mainly composed of vegetation significant greening areas (43.65%). In Xinjiang, the AVHRR data are predominantly vegetation non-significant browning (46.37%), while the MODIS data are mostly vegetation non-significant greening with 43.70%. In the Tibetan Plateau, the vegetation trend with the MODIS data is dominated by total vegetation greening, which is much larger than that of the AVHRR data, accounting for 81.76% and 47.31%, respectively. Except for the Loess Plateau which is dominated by vegetation significant greening and the Northeast region which is mainly vegetation non-significant greening, the other typical regions are all dominated by vegetation browning with AVHRR data and vegetation greening with MODIS data. In the Loess Plateau and Northeast China, the consistent non-significant greening areas are the highest, followed by significant greening, non-significant browning, and significant browning. In Central China and Hebei, Xinjiang, and the Tibetan Plateau, the consistent non-significant greening areas are the highest, followed by non-significant browning, significant greening, and significant browning.

The scatter density map between greening and browning areas detected by AVHRR or MODIS with Landsat was illustrated, showing that the consistent area percentage was 65.71% for Landsat and AVHRR but changed to 84.37% for Landsat and MODIS (**Figure S7**). The inconsistent areas between AVHRR and Landsat accounted for 34.29% of the study area. For the inconsistent regions, areas detected as browning by AVHRR but as greening by Landsat accounted for 29.11%. The inconsistent areas between MODIS and Landsat accounted for 15.62% of the study area. For the inconsistent regions, areas with browning for MODIS but greening for Landsat accounted for 10.28%. The performance of the MODIS NDVI dataset was better than that of any AVHRR–NDVI dataset, which was consistent with the opinion of a previous study (Beck et al., 2011).

## 5 Conclusions

This study used the three common datasets with various spatial resolutions on the Google Earth Engine platform to analyze the vegetation dynamics, and their performances were compared in detecting vegetation greenness of Chinese drylands from 2000 to 2020:

1) Vegetation greening trends were detected from 2000 to 2020 using all NDVI datasets of AVHRR, MODIS, and Landsat in Chinese drylands, especially in the Loess Plateau and Northeast China with significant greening.
2) The vegetation greening areas identified by Landsat comprised more small patches than those by MODIS and AVHRR. The greening areas identified by AVHRR were smaller than those by MODIS and Landsat, while the browning areas identified by AVHRR were larger than those by MODIS and Landsat.
3) Regarding identifying vegetation change, the consistency between Landsat and MODIS data was higher than that of AVHRR. The three datasets exhibited high consistency in identifying vegetation greening in the Northeast, Loess Plateau, and Xinjiang. The areas of inconsistent trends among the three datasets were mainly distributed in the southern part of the Tibetan Plateau, the northern part of North China, South Central China, and Hebei Province.

The research findings can deepen our knowledge on vegetation greenness dynamics in Chinese drylands under the warming-wetting climate trends. Accurately exploring vegetation greening has great significance for achieving sustainable development and measuring the regional carbon budget. Future studies can explore the spatial patterns of vegetation greening with greater precision at finer spatiotemporal scales.

## Author contributions

JZ: methodology, visualization, writing – original draft. YZ and GZ: Conceptualization, Investigation, Writing – review & editing. NC: Project support. ZZ, NC, LT: Data curation. ZZheng: Supervision. GZ, NC, JG, YXZ, and YZ: Resources. All authors contributed to the article and approved the submitted version.
