# Supplementary material for: Coarse spatial resolution remote sensing data with AVHRR and MODIS miss the greening area compared with the Landsat data in Chinese drylands
Source: Front Plant Sci. 2023 May 17;14:1129665. doi: 10.3389/fpls.2023.1129665 (PMC10230077; doi:10.3389/fpls.2023.1129665)
Supplement: Supplementary file 1 [file DataSheet_1.pdf]

Coarse spatial resolution remote sensing data with AVHRR and MODIS misses greening area compared with Landsat data in Chinese drylands

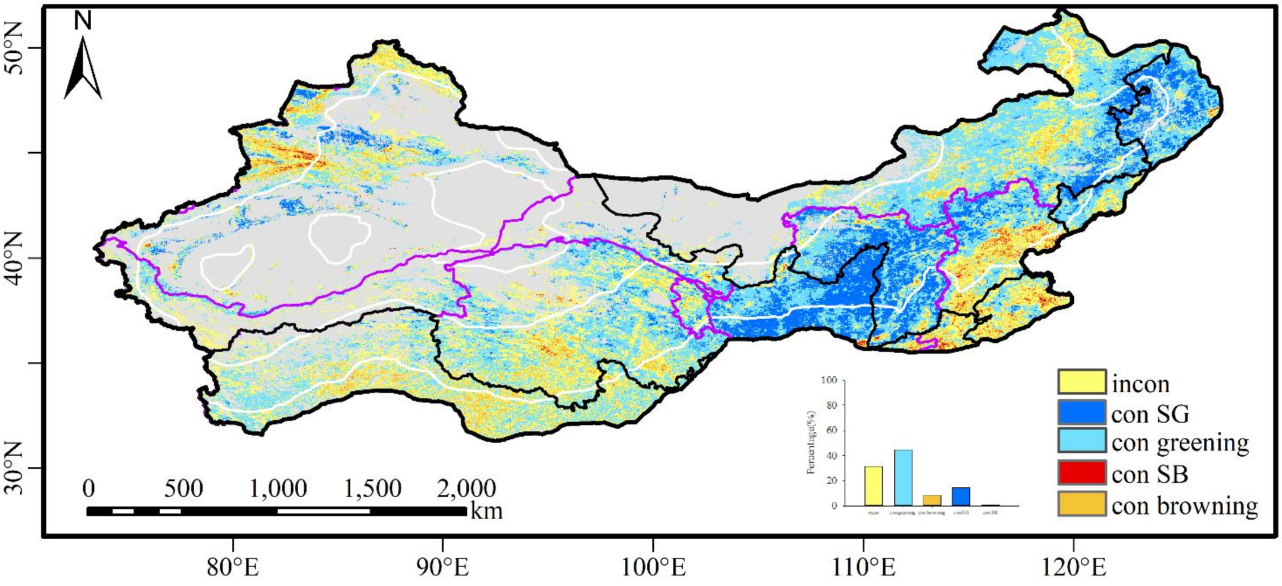

Figure. S1. The spatial distribution of consistent and inconsistent regions based on simultaneous AVHRR and MODIS NDVI datasets from 2000 to 2020.

Note：The consistent vegetation greening areas included the significant greening and non significant greening areas with AVHRR and MODIS, such as the non significant greening with AVHRR and significant greening with MODIS, the significant greening with AVHRR and non significant greening with MODIS, and so did the vegetation browning areas.

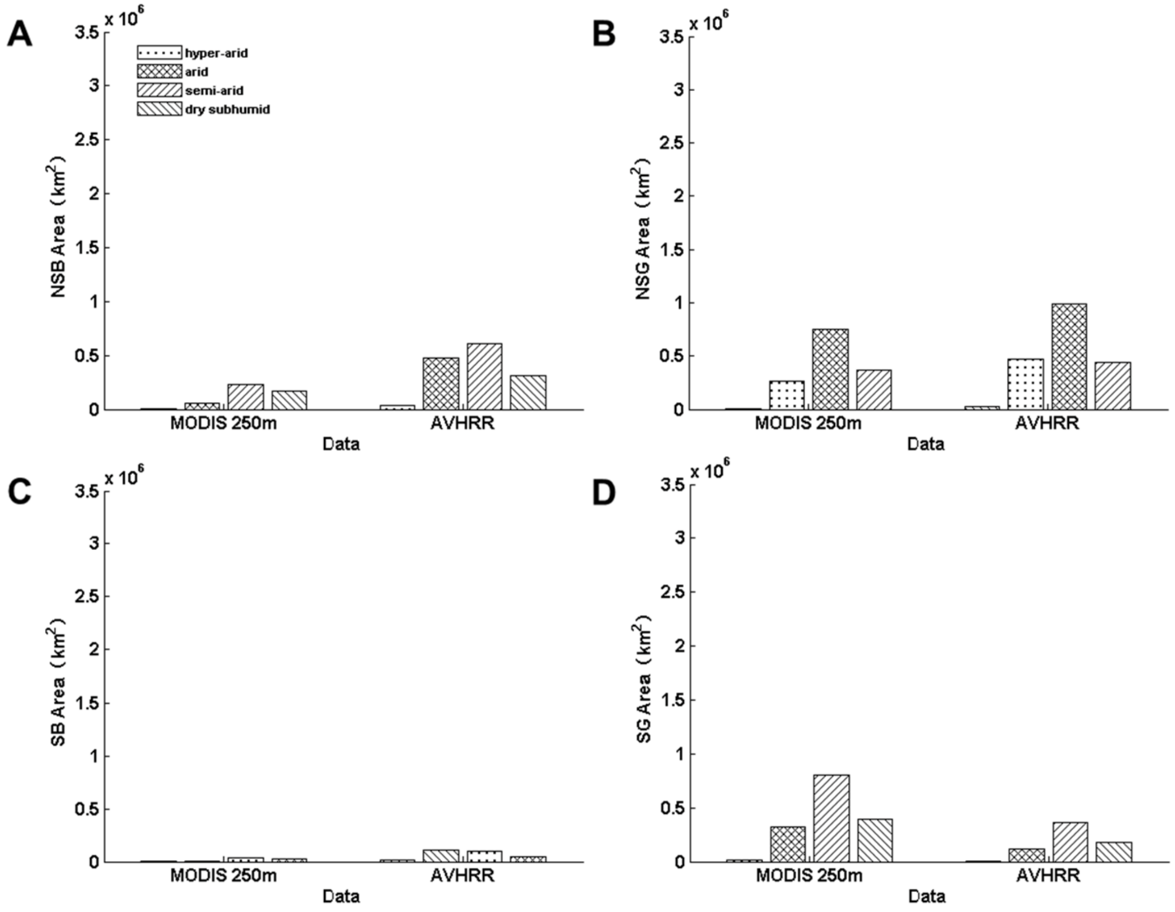

Figure. S2. Vegetation change areas with the two different data from 2000 to 2020 by each dryland type.

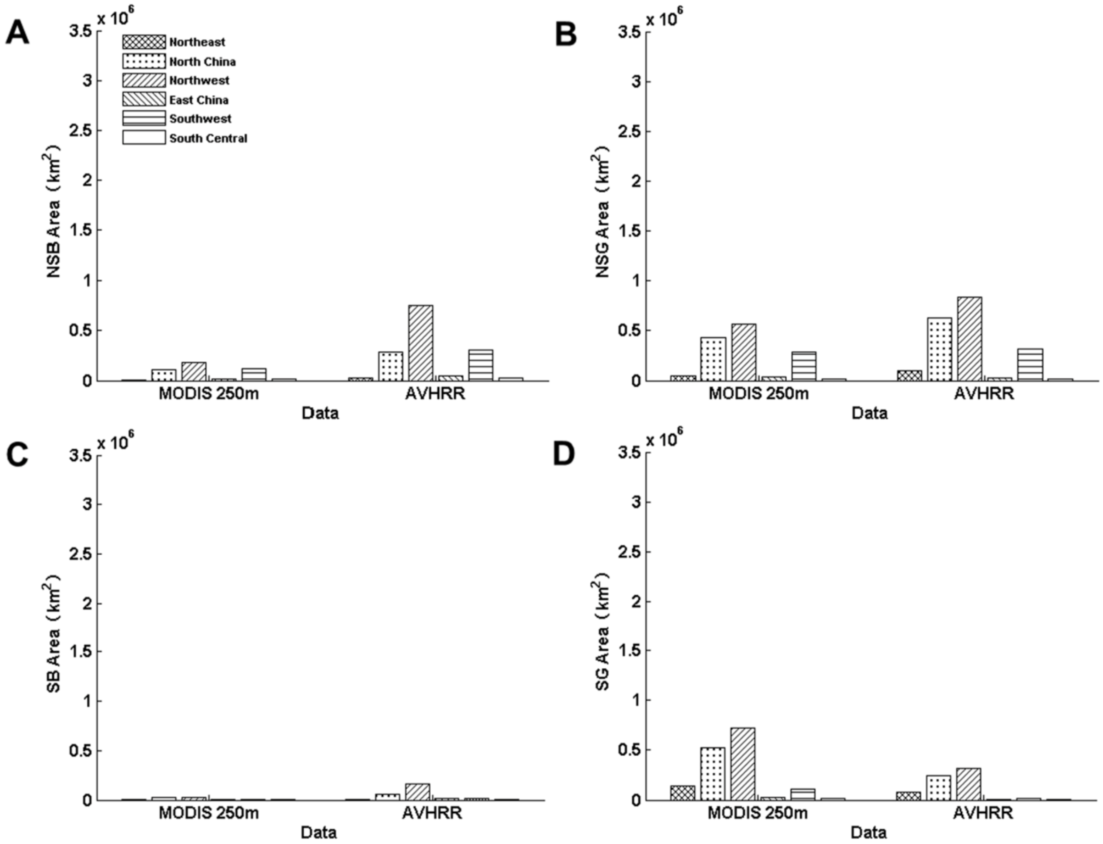

Figure. S3. Vegetation change areas identified with the two datasets from 2000 to 2020 by geographical regions.

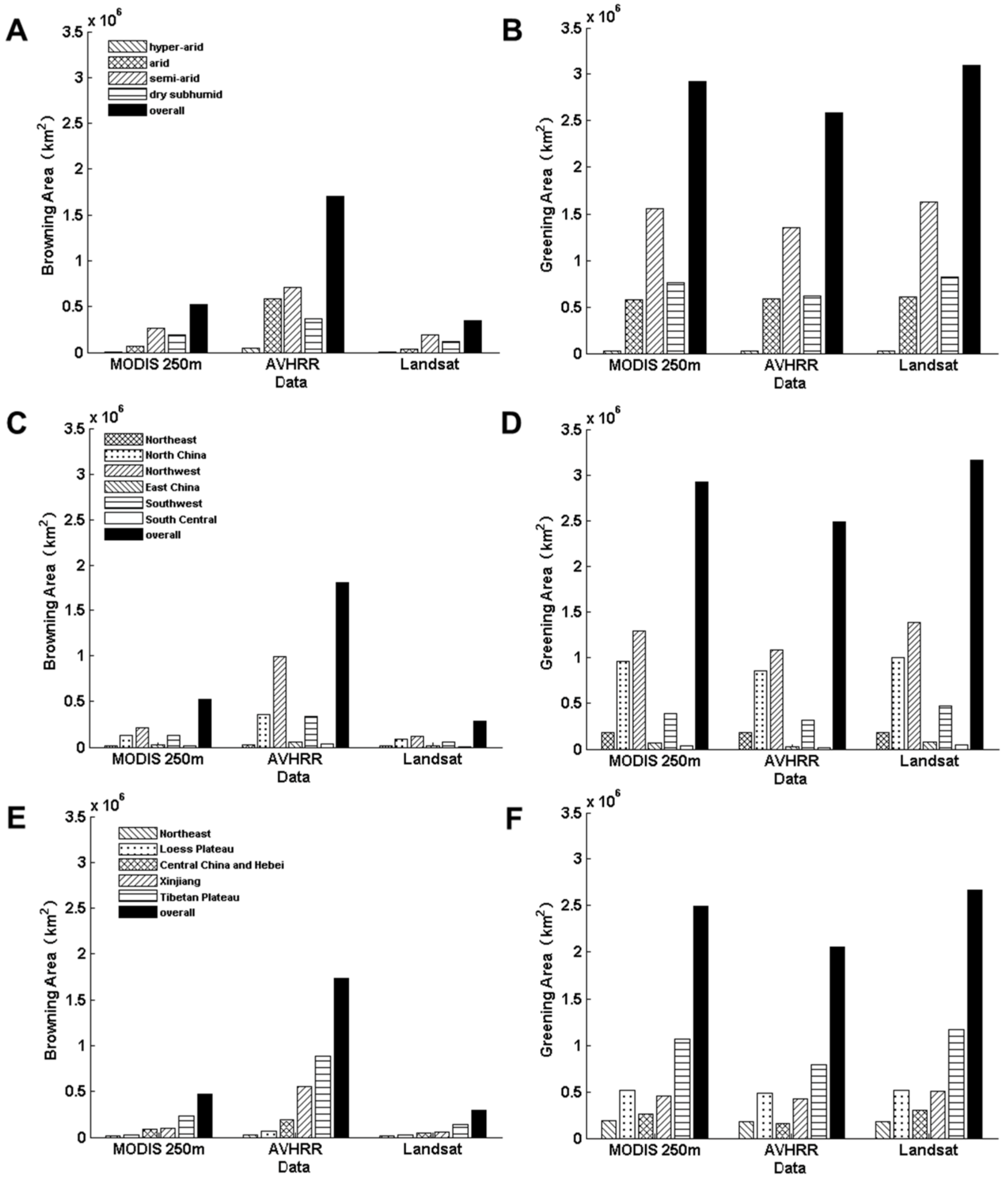

Figure. S4. The vegetation greening and browning areas with MODIS, AVHRR, Landsat data from 2000 to 2020. (A)dryland types (B)geographical regions (C)typical regions.

Note：The vegetation greening areas in the statistics histogram contained both the significant and non-significant, so did the vegetation browning areas.

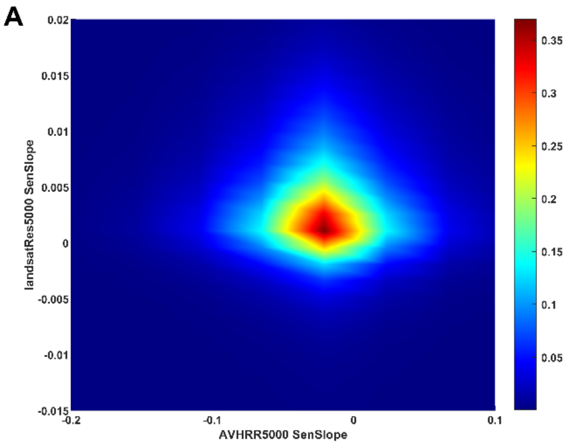

Figure. S5. Scatter density map with sen'slope of the two datasets with Landsat resampled data.

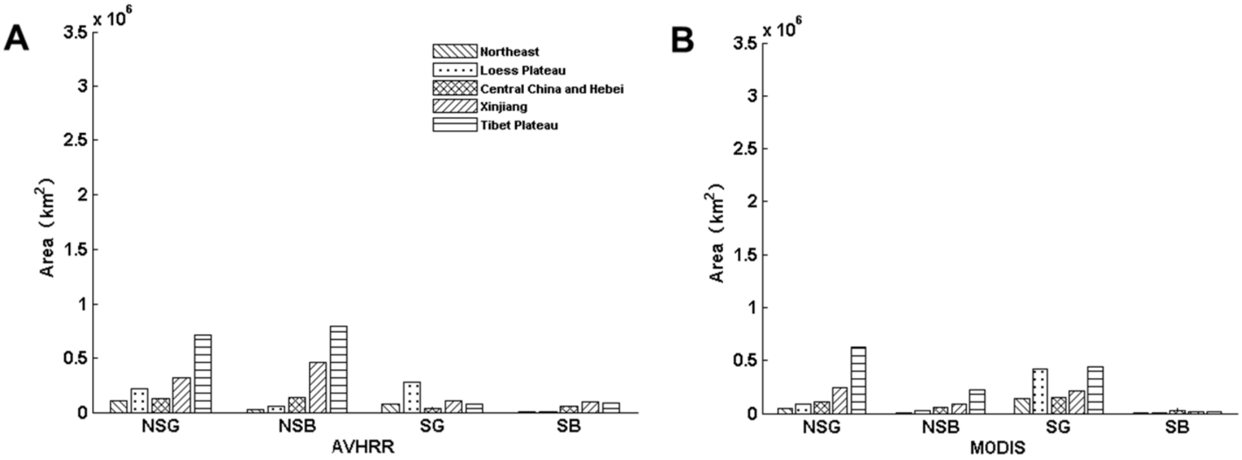

Figure. S6. Statistics histogram of vegetation change areas with the two different datasets (AVHRR and MODIS) from 2000 to 2020, divided by typical regions.

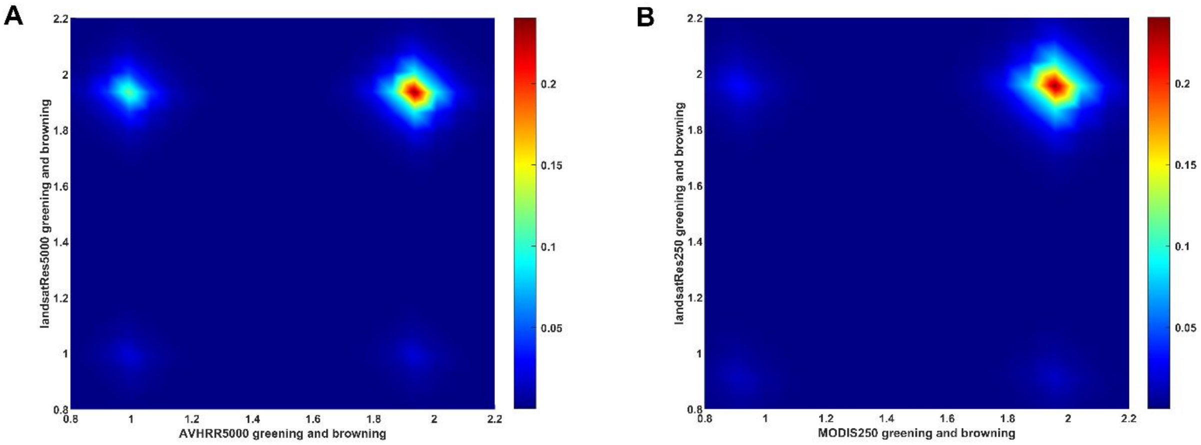

Figure. S7. Scatter density map with greening and browning by MODIS and AVHRR with Landsat data.

Note: Greening was 2, and browning was 1. The consistent regions with Landsat data are the regions where 1 corresponds to 1, and 2 corresponds to 2; The inconsistent regions with Landsat data are the areas where 1 corresponds to 2, and 2 corresponds to 1.
